# Supplementary material for: SMAD2/3 signaling in the uterine epithelium controls endometrial cell homeostasis and regeneration
Source: Commun Biol. 2023 Mar 11;6:261. doi: 10.1038/s42003-023-04619-2 (PMC10008566; doi:10.1038/s42003-023-04619-2)
Supplement: Supplementary file 2 — Supplementary Information [file 42003_2023_4619_MOESM2_ESM.pdf]

SUPPLEMENTAL FILES AND TABLES

SMAD2/3 signaling in the uterine epithelium controls endometrial cell homeostasis and regeneration

Maya L. Kriseman[a,b,c], Suni Tang[a,b], Zian Liao[a,b,d], Peixin Jiang[a,e], Sydney E. Parks[a,b,f], Dominique I. Cope[a,b], Fei Yuan[a,b], Fengju Chen[g,h], Ramya P. Masand[a], Patricia D. Castro[a], Michael M. Ittmann[a], Chad J. Creighton[g,h], Zhi Tan[a,b], and Diana Monsivais[a,b,f,h*]

[a]Department of Pathology and Immunology, Baylor College of Medicine, Houston, TX 77030
[b]Center for Drug Discovery, Baylor College of Medicine, Houston, TX 77030
[c]Division of Reproductive Endocrinology and Infertility, Baylor College of Medicine, Houston, TX 77030
[d]Department of Molecular and Human Genetics, Baylor College of Medicine, Houston, TX 77030
[e]Department of Thoracic/Head and Neck Medical Oncology, the University of Texas MD Anderson Cancer Center, Houston, TX 77030
[f]Cancer and Cell Biology Program, Baylor College of Medicine, Houston, TX, 77030
[g]Department of Medicine, Baylor College of Medicine, Houston, TX 77030
[h]Dan L. Duncan Comprehensive Cancer Center, Baylor College of Medicine, Houston, TX 77030

Supplementary Fig. 1

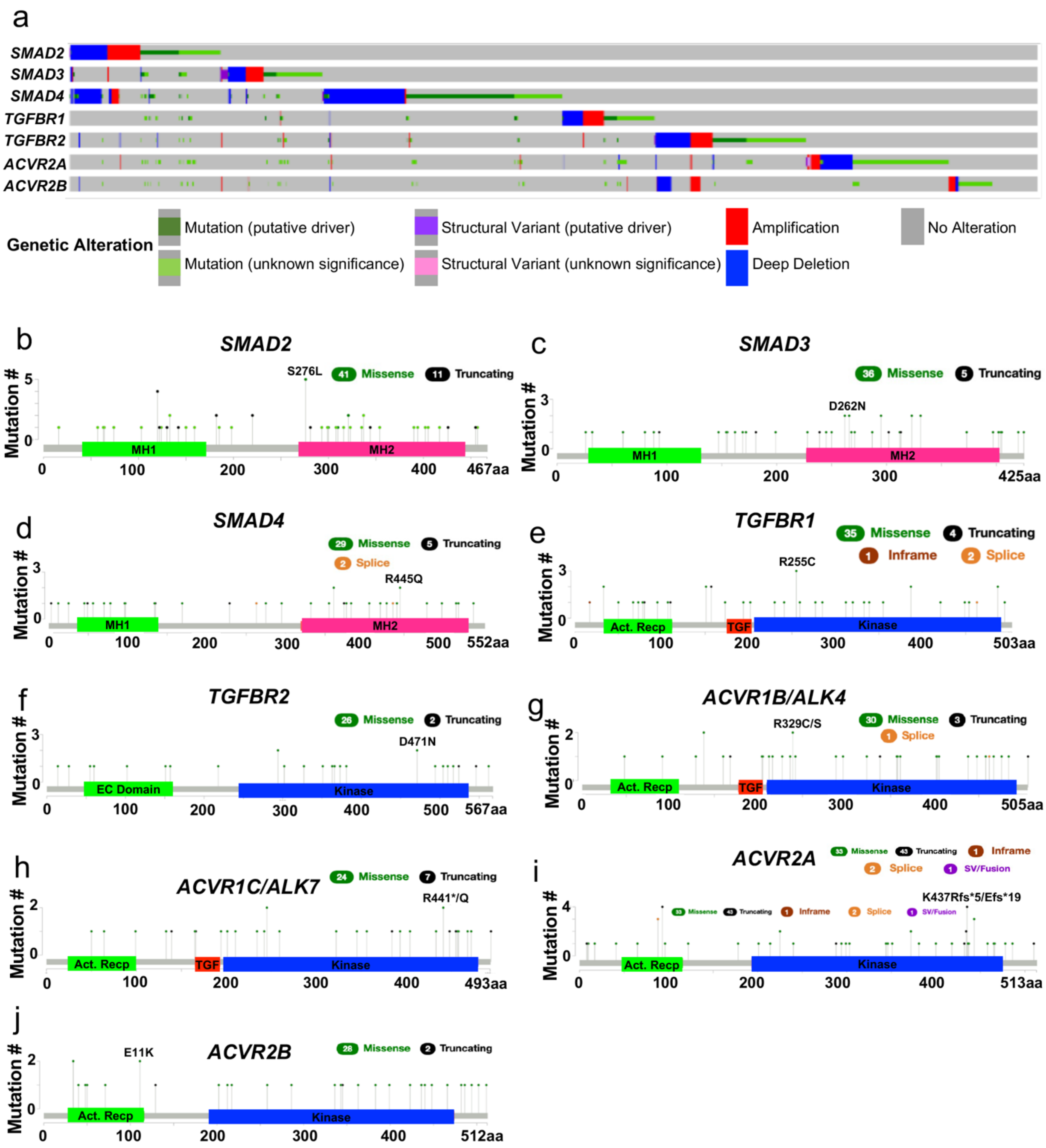

Supplementary Fig. 1: Mutations in the TGFβ signaling pathway in endometrial tumors. A dataset of 894 patients was queried for the presence of mutations in the TGFβ signaling pathway (**a**). This figure shows an overview of the mutations found in the coding regions for *SMAD2, SMAD3, SMAD4* (**b-d**), and in the various TGFβ receptors (**e-j**) that can activate SMAD2/3 signaling. Data represent analysis of 894 patients from the cBioPortal consortium. **b-j** Individual mutations for each of the transcription factors, *SMAD2, SMAD3, SMAD4* and TGFβ receptors, *TGFBR1, TFGBR2, ACVR1B, ACVR1C, ACVR2A,* and *ACVR2B*. The most frequent mutation is noted as well as the predicted effect of the mutation (missense, truncating, in-frame, etc).

Supplementary Fig. 2

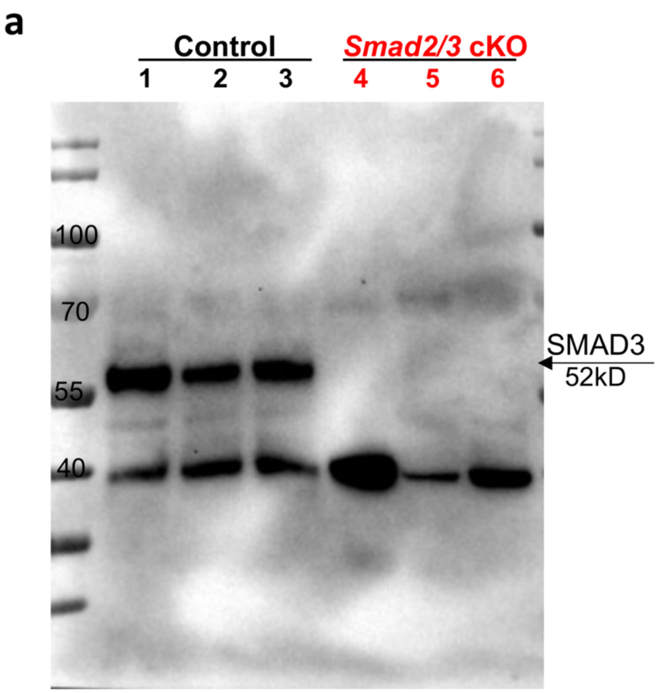

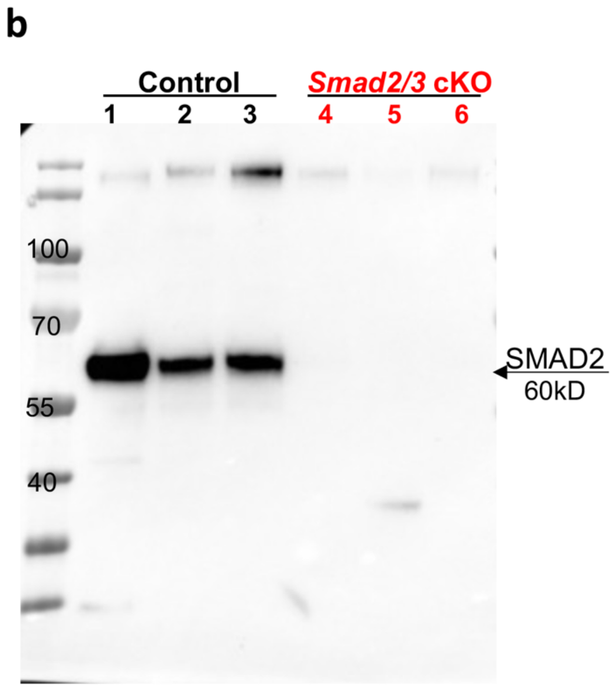

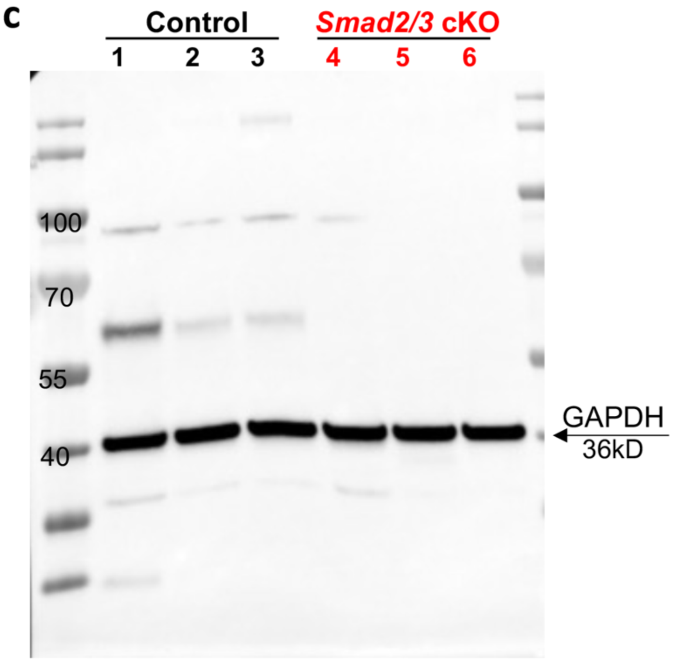

Supplementary Fig. 2: Uncropped western blot images for Fig. 1d. Western blot images for SMAD3 (**a**), SMAD2 (**b**), and GAPDH (**c**), respectively.

Supplementary Fig. 3

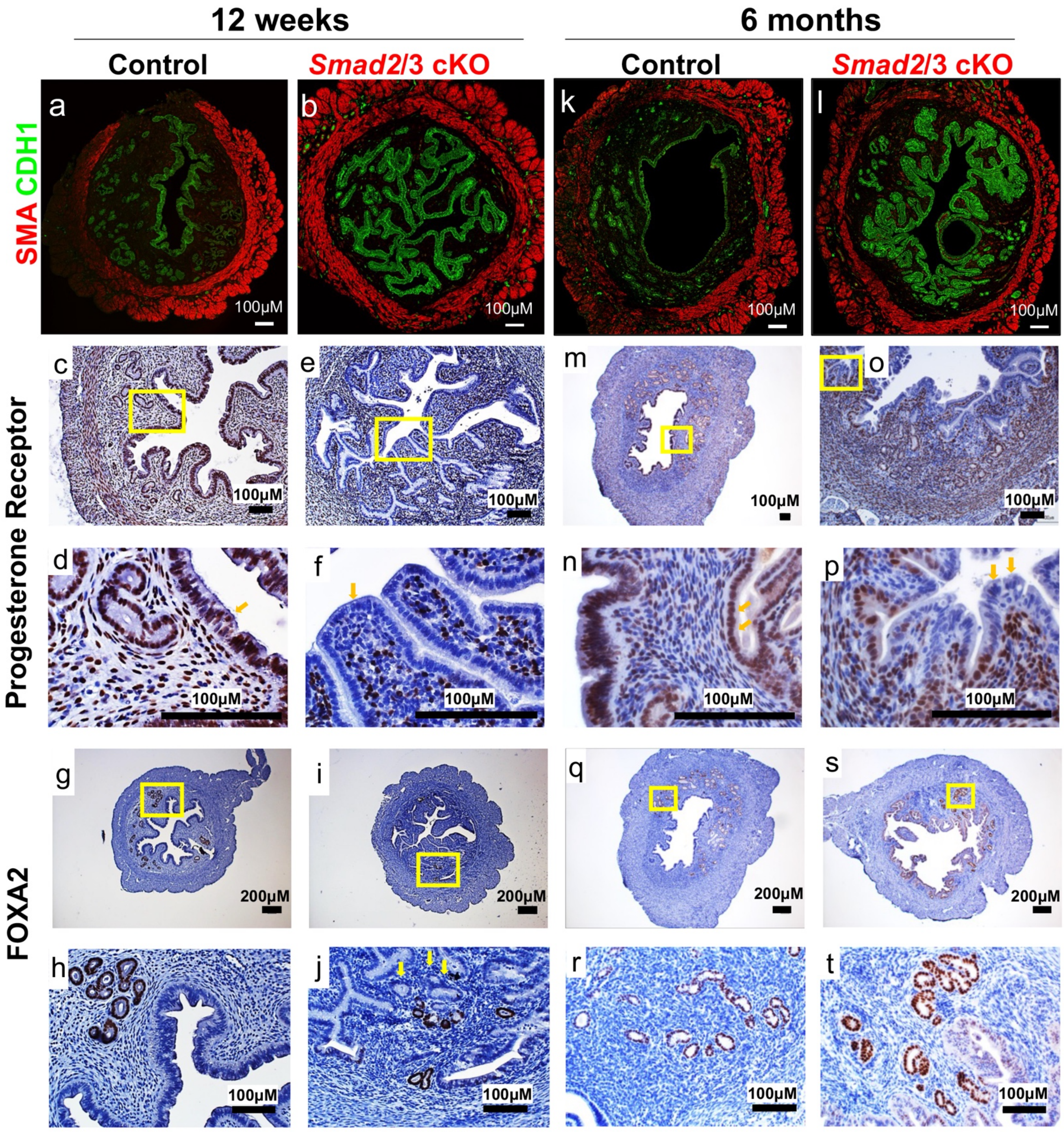

Supplementary Fig. 3: Mice with double SMAD2/3 conditional deletion develop hyperplasia and lose progesterone receptor expression. Uterine cross-sections from 12-week-old mice (**a**,**b**) and 6-month-old mice (**k**, **l**) stained with the myometrial marker, smooth muscle actin (SMA, red) and the epithelial cell marker, E-cadherin (CDH1, green) in control (**a**, **k**) and *Smad2/3* cKO (**b**, **l**) mice. E-

cadherin staining shows that hyperplasia is detected in the uteri of *Smad2/3* cKO mice starting at 12-weeks of age and worsening by 6-months of age. **c-f**, **m-p** Progesterone receptor (PR) immunohistochemistry (IHC) in uterine cross-sections from 12-week-old (**c-f**) and 6-month-old (**m-p**) mice. Results show that compared to controls (**c-d**, **m-n**), *Smad2/3* cKO mice (**e-f**, **o-p**) had decreased PR levels in the uterine epithelium (indicated by yellow arrows in **d**, **f**, **n**, **p**). **g-j**, **q-t** Uterine cross sections from control (**g-h**, **q-r**) and *Smad2/3* cKO mice (**i-j**, **s-t**) showing that FOXA2 expression is expressed in the uterine glands of both genotypes at 12 weeks (**g-j**) and 6 months of age (**q-t**).

Supplementary Fig. 4

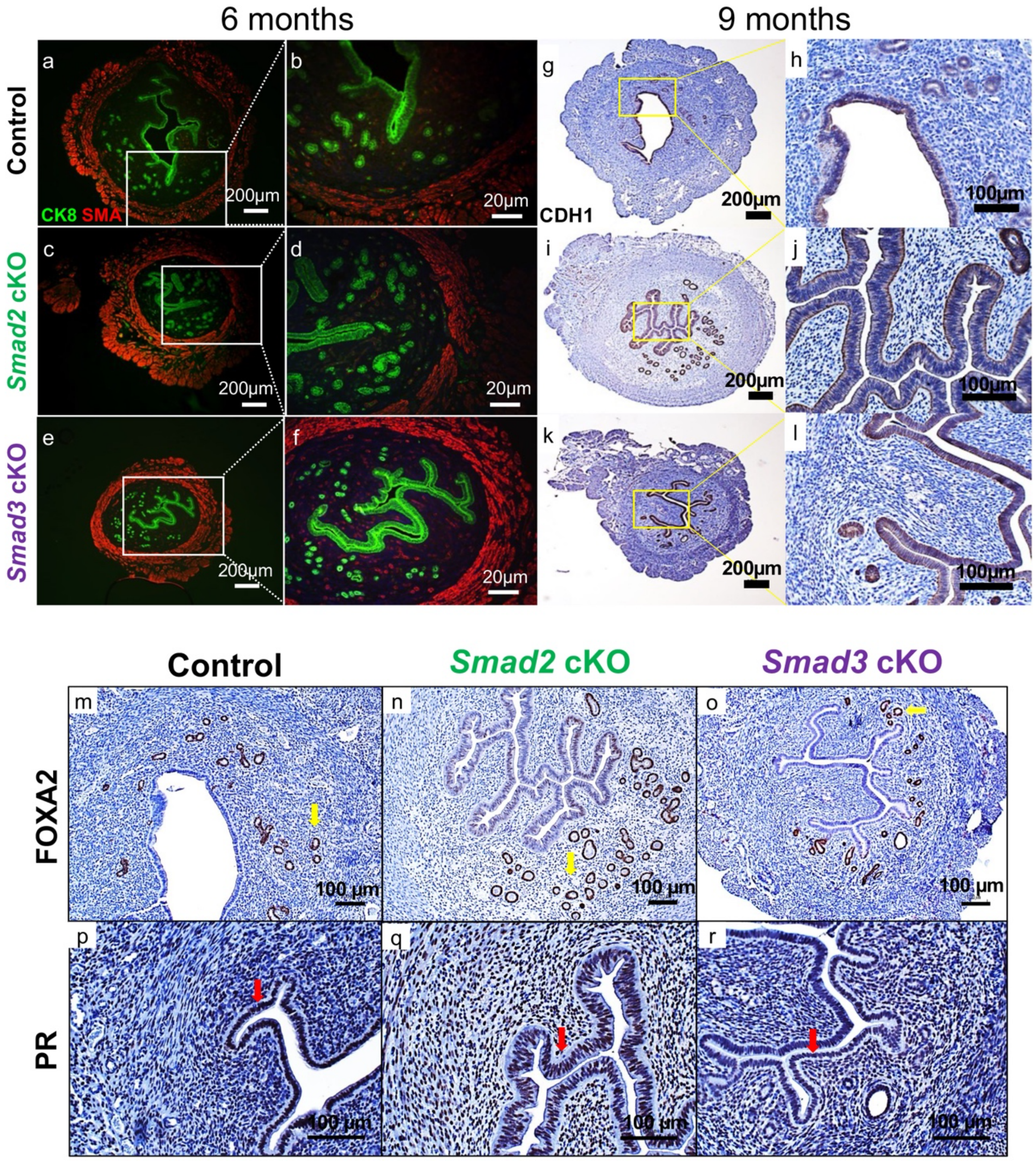

Supplementary Fig. 4: Morphological analysis of uteri from control and single *Smad2* cKO and *Smad3* cKO mice. a-f Cross sections of 6-month-old virgin mice from control (**a-b**), *Smad2* cKO (**c-d**), and *Smad3* cKO (**e-f**) mice stained with cytokeratin 8 (CK8, green) and smooth muscle actin (SMA, red). **g-i** Histological analysis of 9-month-old control (**g-h**), *Smad2* cKO (**i-j**), and *Smad3* cKO (**k-l**) stained with E-cadherin. Mice had been mated during 6 months as part of a breeding trial. **m-n** Uterine cross sections of control (**m**), *Smad2* cKO (**n**), and *Smad3* cKO (**o**) mice stained with the glandular cell marker, FOXA2. **p-r** Uterine cross-sections from control (**p**), *Smad2* cKO (**q**) and *Smad3* cKO (**r**) mice stained with progesterone receptor (PR) antibody.

Supplementary Fig. 5

a

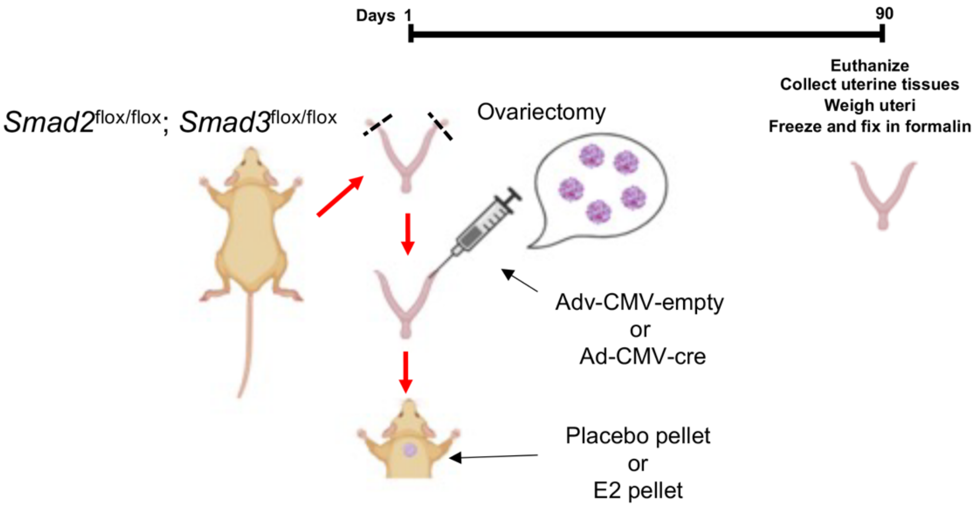

b

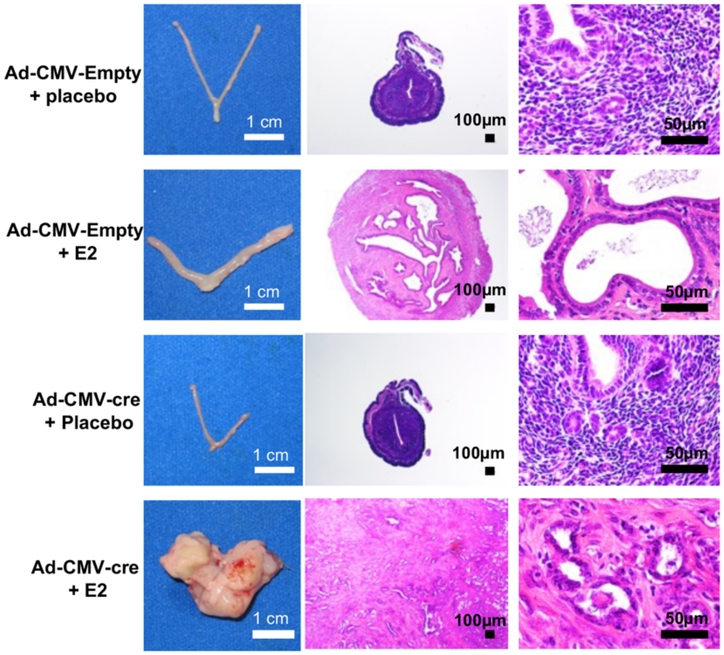

Supplementary Fig. 5: Adenoviral-cre mediated SMAD2/3 deletion in mice. a Diagram showing the schematic used to obtain adenoviral cre-mediated deletion of SMAD2 and SMAD3 in *Smad2*$^{flox/flox}$;*Smad3*$^{flox/flox}$ mice by injection into the uterus of ovariectomized mice treated with a placebo or estradiol (E2) releasing pellet. **b** Analysis of the reproductive tracts 3-months after Ad-cre injection and placebo or E2 treatment. Administration of Ad-cre + E2 led to tumor development, while Ad-cre administration without E2 did not results in endometrial tumors. Experiments were performed in 2-3 mice per genotype.

Supplementary Fig. 6

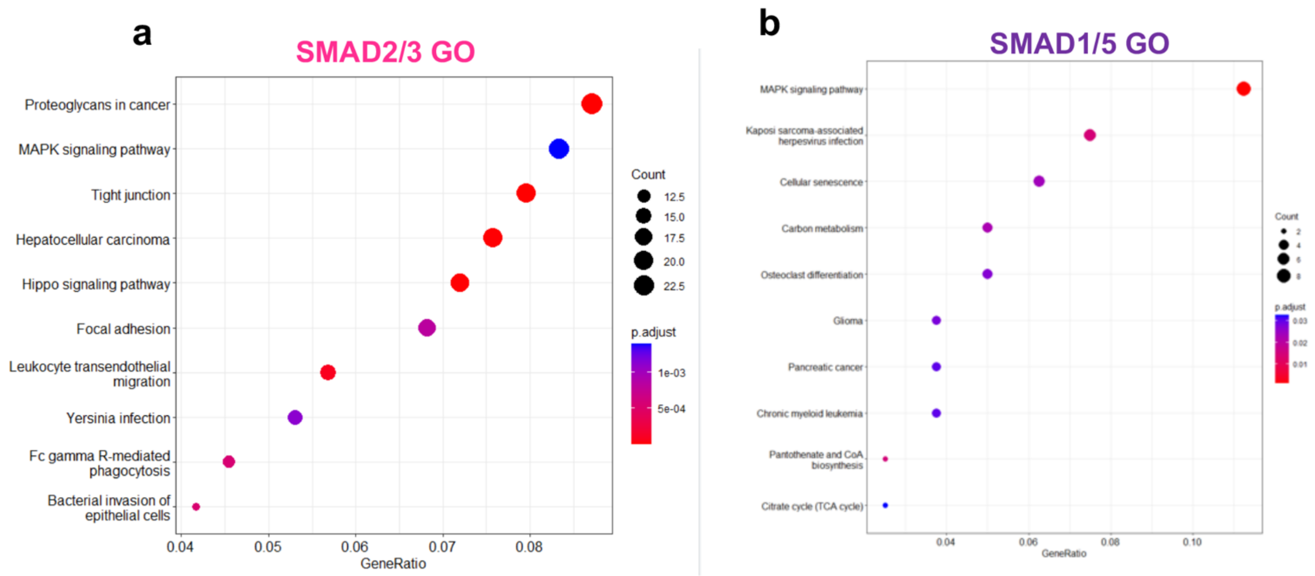

Supplementary Fig. 6: Gene ontology classification in SMAD4-bound genes in endometrial organoids. a-b Gene ontology classifications in the 607 SMAD4-bound genes downregulated in control organoids (representative of SMAD2/3 direct target genes, **a**), and in the 185 SMAD4-bound genes upregulated in *Smad2/3* cKO organoids (representing SMAD1/5 target genes, **b**).

Supplementary Table 1. Development of tumors from *Smad2/3* cKO mice is E2-dependent.

**Treatment**	**Genotype**	**Uterine Weight (g)**	**Uterine tumor**	**Lung Mets**
**No E2**	**Control (n=4)**	**0.0185 ± 0.0034**	**0/4**	**0/4**
	***Smad2/3* cKO (n=4)**	**0.025 ± 0.002**	**0/4**	**0/4**
**E2 pellet**	**Control (n=4)**	**0.1079 ± 0.0382**	**0/4**	**0/4**
	***Smad2/3* cKO (n=4)**	**2.99 ± 0.66**	**4/4**	**2/4**

Supplementary Table 2. Primer sequences used for genotyping and quantitative PCR.

Gene	Primer #	Sequence (5'-3')
Smad2	F	TACTTGGGGCAATCTTTTCG
Genotyping	R	GTCACTCCCTGAACCTGAAG
Smad3	F	CTCCAGATCGTGGGCATACAGC
Genotyping	R	GGTCACAGGGTCCTCTGTGCC
Ltf-cre	1	GTTTCCTCCTTCTGGGCTCC
	2	TTTAGTGCCCAGCTTCCCAG
	3	CCTGTTGTTCAGCTTGCACC
Aldh1a1	F	ATACTTGTCGGATTTAGGAGGCT
	R	GGGCCTATCTTCCAAATGAACA
Aldh1a2	F	CAGAGAGTGGGAGAGTGTTCC
	R	CACACAGAACCAAGAGAGAAGG
Aldh1a3	F	GGGTCACACTGGAGCTAGGA
	R	CTGGCCTCTTCTTGGCGAA
Cyp26a1	F	AAGCTCTGGGACCTGTACTGT
	R	CTCCGCTGAAGCACCATCT
Lrat	F	CCGTCCCTATGAAATCAGCTC
	R	ATGGGCGACACGGTTTTCC
Rbp4	F	CCACTGGATCATCGACACGG
	R	GCCATTGGGGTCACGAGAA
Id1	F	CCTAGCTGTTCGCTGAAGGC
	R	CTCCGACAGACCAAGTACCAC

Id2	F	ATGAAAGCCTTCAGTCCGGTG
	R	AGCAGACTCATCGGGTCGT
Id3	F	CGACCGAGGAGCCTCTTAG
	R	GGACGCGATAGGGAAGACC
Id4	F	ATGAAAGCCTTCAGTCCGGTG
	R	AGCAGACTCATCGGGTCGT
Smad2	F	GCTCTTCTGGCTCAGTCTGTCA
Exon 10	R	GGTGCACATTCGGGTTAGCT
Smad3	F	TCACGTTATCTACTGCCGCC
Exon 3	R	AGCTCCATGGCCCGTAATTC

Supplementary Table 3. Antibody information.

Antigen	Source	Cat. No.	Dilution
SMAD2	Cell Signaling	5339	WB 1:1000
SMAD3	Cell Signaling	9523	WB 1:1000
pSMAD2	Cell Signaling	3108S	WB 1:200
pSMAD2/3	Cell Signaling	8828S	IHC 1:100
SMA	Abcam	Ab5694	IHC 1:500
Progesterone Receptor	Cell Signaling	8757	IHC 1:200
FOXA2	Abcam	ab108422	IHC/IF 1:200
ERα	Cell Signaling	13258S	IHC 1:200
TTF1	Abcam	Ab76013	IHC, IF 1:200
CDH1	Cell Signaling	3195S	IHC: 1:200
CK8	DSHB	TROMA-I	IF 1:50
MUC1	Novusbio	NB120-15481	IHC 1:200
SMAD4	Abcam	Ab40759	0.678μg/reaction
ALDH1A1	Abcam	Ab52492	IHC 1:50
ALDH1A2	Sigma	HPA010022	IHC 1:500
ALDH1A3	GeneTex	GTX110784	IHC 1:100
pSMAD1/5	Cell Signaling	9516	IHC 1:200

Supplementary Data Files

Supplementary Data 1. Gene ontology analysis of differentially expressed genes in endometrial organoids from control, control + A83-01, and *Smad2/3* cKO mice. *Attached as an excel spreadsheet.*

Supplementary Data 2. SMAD4 bound genes that are up- or down-regulated in RNAseq datasets of control and *Smad2/3* cKO organoids. 607 SMAD4-bound genes downregulated in *Smad2/3* cKO vs control organoids (representing SMAD2/3 target genes) and 185 SMAD4-bound genes upregulated in *Smad2/3* cKO vs. control organoids (representing SMAD1/5 target genes). *Attached as an excel spreadsheet.*

Supplementary Data 3. The source data behind the graphs in the paper. *Attached as an excel spreadsheet.*
