# Supplementary material for: SMAD2/3 signaling in the uterine epithelium controls endometrial cell homeostasis and regeneration
Source: Commun Biol. 2023 Mar 11;6:261. doi: 10.1038/s42003-023-04619-2 (PMC10008566; doi:10.1038/s42003-023-04619-2)
Supplement: Supplementary file 3 — Description of Additional Supplementary Files [file 42003_2023_4619_MOESM3_ESM.pdf]

Description of Additional Supplementary Files

File name: Supplementary Data 1.
Description: Gene ontology analysis of differentially expressed genes in endometrial organoids from control, control + A83-01, and Smad2/3 cKO mice. Attached as an excel spreadsheet.

File name: Supplementary Data 2.
Description: SMAD4 bound genes that are up- or down-regulated in RNAseq datasets of control and Smad2/3 cKO organoids. 607 SMAD4-bound genes downregulated in Smad2/3 cKO vs control organoids (representing SMAD2/3 target genes) and 185 SMAD4-bound genes upregulated in Smad2/3 cKO vs. control organoids (representing SMAD1/5 target genes). Attached as an excel spreadsheet.

File name: Supplementary Data 3.
Description: The source data behind the graphs in the paper. Attached as an excel spreadsheet.
