# Supplementary material for: SMAD2/3 signaling in the uterine epithelium controls endometrial cell homeostasis and regeneration
Source: Commun Biol. 2023 Mar 11;6:261. doi: 10.1038/s42003-023-04619-2 (PMC10008566; doi:10.1038/s42003-023-04619-2)
Supplement: Supplementary file 7 — Reporting Summary [file 42003_2023_4619_MOESM7_ESM.pdf]

nature portfolio

Corresponding author(s): Diana Monsivais

Last updated by author(s): Jan 25, 2023

Reporting Summary

Nature Portfolio wishes to improve the reproducibility of the work that we publish. This form provides structure for consistency and transparency in reporting. For further information on Nature Portfolio policies, see our Editorial Policies and the Editorial Policy Checklist.

Statistics

For all statistical analyses, confirm that the following items are present in the figure legend, table legend, main text, or Methods section.

n/a	Confirmed	
☐	☒	The exact sample size (*n*) for each experimental group/condition, given as a discrete number and unit of measurement
☐	☒	A statement on whether measurements were taken from distinct samples or whether the same sample was measured repeatedly
☐	☒	The statistical test(s) used AND whether they are one- or two-sided<br>*Only common tests should be described solely by name; describe more complex techniques in the Methods section.*
☐	☒	A description of all covariates tested
☐	☒	A description of any assumptions or corrections, such as tests of normality and adjustment for multiple comparisons
☐	☒	A full description of the statistical parameters including central tendency (e.g. means) or other basic estimates (e.g. regression coefficient) AND variation (e.g. standard deviation) or associated estimates of uncertainty (e.g. confidence intervals)
☐	☒	For null hypothesis testing, the test statistic (e.g. *F*, *t*, *r*) with confidence intervals, effect sizes, degrees of freedom and *P* value noted<br>*Give P values as exact values whenever suitable.*
☒	☐	For Bayesian analysis, information on the choice of priors and Markov chain Monte Carlo settings
☒	☐	For hierarchical and complex designs, identification of the appropriate level for tests and full reporting of outcomes
☒	☐	Estimates of effect sizes (e.g. Cohen's *d*, Pearson's *r*), indicating how they were calculated

Our web collection on statistics for biologists contains articles on many of the points above.

Software and code

Policy information about availability of computer code

Data collection

RNA Sequencing data are available in the Gene Expression Omnibus (GSE212475).
CUT and RUN: Sequencing data are available in the Gene Expression Omnibus (GSE212474).

Data analysis

RNAseq: Reads were filtered, trimmed, and aligned to the mouse genome (build GRCm39) using Slamon 1.4.0. Differentially expressed genes were calculated using DEseq2 (version 1.32.0) with fold chance > 1.4 and < 0.714 and adjusted p-value less than 0.01 and visualized using ggplot2 (version 3.3.5). Gene ontologies of genes classified to be up- or downregulated were obtained using Sigterms v169, adjusted p-values and visualizations were created using enrichplot (R package version 1.16.1)70.

CUT and RUN: Raw data were de-multiplexed by bcl2fastq v2.20 with fastqc for quality control. Clean reads were mapped to reference genome mm10 by Bowtie2 (v2.2.7), with parameters of --end-to-end --very-sensitive --no-mixed --no-discordant --phred33 -I 10 -X 700. For Spike-in mapping, reads were mapped to E. coli genome U00096.3. Duplicated reads were removed, and only uniquely mapped reads were kept. Spike-in normalization was achieved through multiply primary genome coverage by scale factor (100000 / fragments mapped to E. coli genome). CUT&RUN peaks were called by MACS2 (v2.1.0) with the parameters of -f BAM -q 0.1 -n. Track visualization was done by bedGraphToBigWig71, bigwig files were imported to Integrative Genomics Viewer for visualization. For peak annotation, common peaks between duplicates were identified with 'mergePeaks' function in homer v4.11 and then genomic annotation was added by ChIPseeker72. Motif analysis was conducted through HOMER v4.11 on the merged peaks with parameter set as findMotifsGenome.pl mm10 -size 200 –mask. Integration of SMAD4 peaks with differentially expressed genes from the RNAseq analysis was performed using DiffBind with FDR<0.0573.

For manuscripts utilizing custom algorithms or software that are central to the research but not yet described in published literature, software must be made available to editors and reviewers. We strongly encourage code deposition in a community repository (e.g. GitHub). See the Nature Portfolio guidelines for submitting code & software for further information.

Data

Policy information about availability of data

All manuscripts must include a data availability statement. This statement should provide the following information, where applicable:

- Accession codes, unique identifiers, or web links for publicly available datasets
- A description of any restrictions on data availability
- For clinical datasets or third party data, please ensure that the statement adheres to our policy

Sequencing analyses are freely available and deposited in the Gene Expression Omnibus under accession number GSE212477 superseries.

Human research participants

Policy information about studies involving human research participants and Sex and Gender in Research.

Reporting on sex and gender	Not applicable
Population characteristics	Not applicable
Recruitment	Not applicable
Ethics oversight	Not applicable

Note that full information on the approval of the study protocol must also be provided in the manuscript.

Field-specific reporting

Please select the one below that is the best fit for your research. If you are not sure, read the appropriate sections before making your selection.

☒ Life sciences ☐ Behavioural & social sciences ☐ Ecological, evolutionary & environmental sciences

For a reference copy of the document with all sections, see nature.com/documents/nr-reporting-summary-flat.pdf

Life sciences study design

All studies must disclose on these points even when the disclosure is negative.

Sample size	For our mouse studies, the sample size was determined based on a power analysis calculation that took into account various paraments, such as effect size, standard deviation, type 1 error and direction of effect. Because of the genetic nature of our animal studies, and the limitations based on sex and age, we also factored an expected attrition of animals in our breeding schemes.
Data exclusions	Not applicable
Replication	To verify the reproducibility of our studies, experiments were repeated in samples from various subjects and performed on different dates, often by different investigators.
Randomization	Samples and organisms were separated into specific groups based on genotype. Because studies were investigating uterine function, only female mice were utilized.
Blinding	Because the analysis was being performed on grouped mice according to genotype, and the differences in gene expression and histology were based on these genetic differences, blinding was not performed.

Reporting for specific materials, systems and methods

We require information from authors about some types of materials, experimental systems and methods used in many studies. Here, indicate whether each material, system or method listed is relevant to your study. If you are not sure if a list item applies to your research, read the appropriate section before selecting a response.

Materials & experimental systems

n/a	Involved in the study	
☐	☒	Antibodies
☒	☐	Eukaryotic cell lines
☒	☐	Palaeontology and archaeology
☐	☒	Animals and other organisms
☒	☐	Clinical data
☒	☐	Dual use research of concern

Methods

n/a	Involved in the study	
☒	☐	ChIP-seq
☒	☐	Flow cytometry
☒	☐	MRI-based neuroimaging

Antibodies

Antibodies used

SMAD2, Cell Signaling #5339, WB 1:1000; SMAD3, Cell Signaling #9523, WB 1:1000, pSMAD2, Cell Signaling, 3108S, WB 1:200; SMA, Abcam #Ab5694, IHC 1:500; Progesterone Receptor, Cell Signaling #8757 IHC 1:200; FOXA2, Abcam #ab108422, IHC/IF 1:200; Erα, Cell Signaling #13258S, IHC 1:200, TTF1, Abcam #Ab76013, IHC, IF 1:200; CDH1, Cell Signaling #3195S, IHC: 1:200; CK8, DSHB TROMA-I, IF 1:50; MUC1, Novusbio #NB120-15481, IHC 1:200; SMAD4, Abcam #Ab40759, 0.678µg/reaction; ALDH1A1, Abcam # Ab52492, IHC 1:50; ALDH1A2, Sigma #HPA010022, IHC 1:500; ALDH1A3, GeneTex #GTX110784, IHC 1:100; pSMAD1/5, Cell Signaling #9516, IHC 1:200

Validation

1) SMAD2, Cell Signaling #5339, WB 1:1000; is validated by the manufacturer, is shown to be highly specific, and has 497 associated references

2) SMAD3, Cell Signaling #9523, WB 1:1000; is validated by the manufacturer for western blot and other applications and has 509 published references

3) pSMAD2, Cell Signaling, 3108S, WB 1:200; is validated by the manufacturer and has 701 references.

4) SMA, Abcam #Ab5694, IHC 1:500; This antibody is validated for various applications by the manufacturer and has 2188 citations in the literature.

5) Progesterone Receptor, Cell Signaling #8757 IHC 1:200; Is a validated antibody by the manufacturer for various applications. The antibody has 72 references.

6) FOXA2, Abcam #ab108422, IHC/IF 1:200; is validated by the manufacturer for various applications and has 65 associated citations.

7) Erα, Cell Signaling #13258S, IHC 1:200; is validated for various applications by the manufacturer and has 28 associated citations.

8) TTF1, Abcam #Ab76013, IHC, IF 1:200; is validated for IHF and Western Blot by the manufacturer and has 110 published citations

9) CDH1, Cell Signaling #3195S, IHC: 1:200; is an antibody validated for various applications and has 2445 citations.

10) CK8, DSHB TROMA-I, IF 1:50; is validated for immunostaining by the manufacturer and has been widely used in the literature for many years.

11) MUC1, Novusbio #NB120-15481, IHC 1:200; this antibody is validate on the manufacturer's website for its use in IHC. It is listed on 10 publications.

12) SMAD4, Abcam #Ab40759, 0.678µg/reaction; SMAD4 antibody has been used for various applications and validated by the manufacturer. It has 136 citations.

13) ALDH1A1, Abcam # Ab52492, IHC 1:50; This antibody is validated for various applications on the website by the manufacturer and has 148 associated references.

14) ALDH1A2, Sigma #HPA010022, IHC 1:500; Is validated for IHC on the website and has been used in 34 publications.

15) ALDH1A3, GeneTex #GTX110784, IHC 1:100; is validated for western blots and cytology on the manufacturer's website and has 3 references.

16) pSMAD1/5, Cell Signaling #9516, IHC 1:200; this product is validated by the manufacturer and has 376 citations.

17)pSMAD2/3, Cell Signaling #8828, IHC 1:100, this product is validated by the manufacturer and has 603 citations, including 12 for IHC.

Animals and other research organisms

Policy information about studies involving animals; ARRIVE guidelines recommended for reporting animal research, and Sex and Gender in Research

Laboratory animals	Mice were maintained on a mixed C57BL/6J and 129S5/SvEvBrd genetic background. Studies were performed in adult mice (6-8 weeks old) unless indicated otherwise.
Wild animals	Not applicable
Reporting on sex	Because we are studying the effect of the SMAD2 and SMAD3 transcription factors in the uterus, only adult mice of the female sex were used for our studies. Males with the required genotype were used to generate the experimental mice.
Field-collected samples	No field-collected samples were used.
Ethics oversight	All mouse handling and experimental studies were performed under protocols approved by the Institutional Animal Care and Use Committee of Baylor College of Medicine and guidelines established by the NIH Guide for the Care and Use of Laboratory Animals.

Note that full information on the approval of the study protocol must also be provided in the manuscript.
